# Transfer of Galectin-3-Binding Protein via Epididymal Extracellular Vesicles Promotes Sperm Fertilizing Ability and Developmental Potential in the Domestic Cat Model

**DOI:** 10.3390/ijms24043077

**Published:** 2023-02-04

**Authors:** Tricia Rowlison, Pierre Comizzoli

**Affiliations:** Smithsonian National Zoo and Conservation Biology Institute, Washington, DC 20008, USA

**Keywords:** galectin-3-binding protein, spermatozoa, epididymis, extracellular vesicle, centrosome

## Abstract

Key proteins transferred by epididymal extracellular vesicles (EVs) to the transiting sperm cells contribute to their centrosomal maturation and developmental potential. Although not reported in sperm cells yet, galectin-3-binding protein (LGALS3BP) is known to regulate centrosomal functions in somatic cells. Using the domestic cat model, the objectives of this study were to (1) detect the presence and characterize the transfer of LGALS3BP via EVs between the epididymis and the maturing sperm cells and (2) demonstrate the impact of LGALS3BP transfer on sperm fertilizing ability and developmental potential. Testicular tissues, epididymides, EVs, and spermatozoa were isolated from adult individuals. For the first time, this protein was detected in EVs secreted by the epididymal epithelium. The percentage of spermatozoa with LGALS3BP in the centrosome region increased as cells progressively incorporated EVs during the epididymal transit. When LGALS3BP was inhibited during in vitro fertilization with mature sperm cells, less fertilized oocytes and slower first cell cycles were observed. When the protein was inhibited in epididymal EVs prior to incubation with sperm cells, poor fertilization success further demonstrated the role of EVs in the transfer of LGALS3BP to the spermatozoa. The key roles of this protein could lead to new approaches to enhance or control fertility in clinical settings.

## 1. Introduction

Mammalian sperm cells undergo numerous modifications of their structure, composition, and function throughout epididymal transit. Many of these changes are essential to subsequent interactions with the female gamete, including the progress through cumulus cells, binding and crossing of the zona pellucida, fusion with the oolemma, and completion of the first cell cycle [1,2,3,4,5]. Importantly, the integration of small RNAs and specific peptides into the sperm cells during epididymal transit contribute to this maturation process. Following fertilization, sperm cells deliver small RNAs regulating post-transcriptional gene expression during the subsequent embryonic development [6,7,8,9,10]. Additionally, proteins integrated into the sperm cells during epididymal transit affect critical functions, such as sperm motility, fertilization, and early embryonic development [1,2,3,11,12,13,14]. In addition to epididymal merocrine secretions of maturation factors directly incorporated into the sperm cells, small RNAs and proteins without a N-terminal signal sequence are secreted by the epididymal epithelium within membranous vesicles termed extracellular vesicles (EV). These vesicles, also called epididymosomes, range in size from approximately 30 to 500 nm and will fuse with the outer plasma membrane for cellular uptake [15,16,17,18] as previously reported in several species, including the cat [19], mouse [18,20], rat [21], hamster [22], man [23], and bull [24].

**Figure 1:**
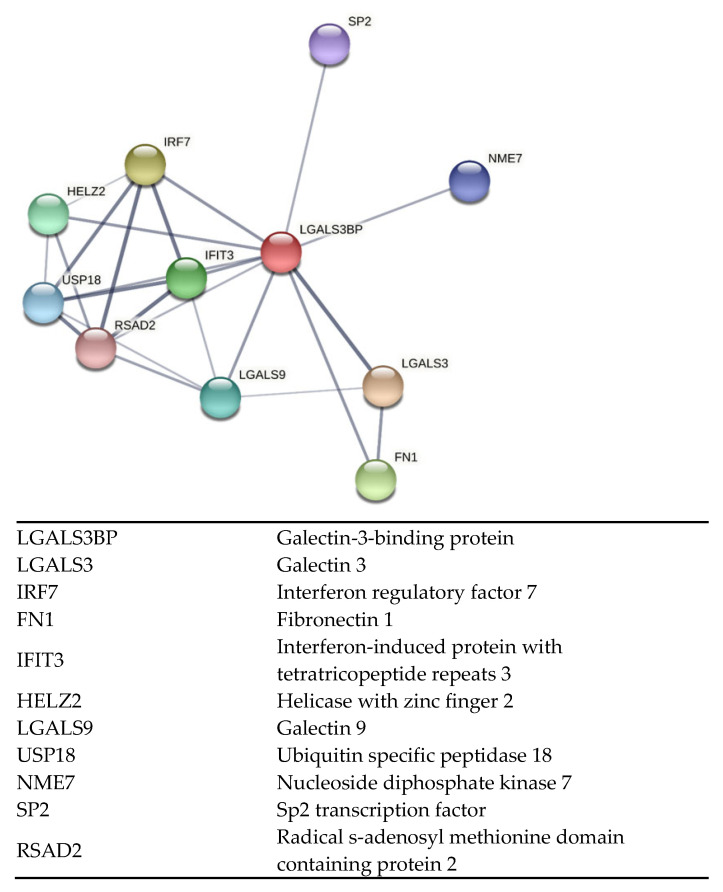
Interactive network of predicted protein–protein associations with LGALS3BP in the domestic cat species. Edges represent both physical and functional protein associations (STRING 11.5).

Former studies in bovine and mouse models have shown that epididymal EV proteins are incorporated by the sperm cells in a sequential manner [1,14]. Using the domestic cat, a critical biomedical model for humans and other animal species, we previously demonstrated that exposing immature sperm cells to EVs improved motility and subsequent embryonic development partly because of the incorporation of key peptides [5,25,26,27]. However, the functional effects of proteins enclosed in epididymal EVs merit further exploration to refine our understanding of sperm maturation in mammals. Specifically, a protein that deserves more attention is the galectin-3-binding protein (LGALS3BP). LGALS3BP is part of the galectin family which is comprised of beta-galactoside-binding proteins [28]. These proteins are primarily implicated in modulating cell–cell and cell–matrix interactions as well as centriole biogenesis [28,29,30]. Post-translational modification of LGALS3BP results in oligomeric ring-like structures largely due to N- and O-glycosylation modifications, which allow binding with numerous factors in the extracellular matrix, including fibronectin, galectins, and integrins [28]. Furthermore, predicted interactions between LGALS3BP with other proteins in the domestic cat indicate its role in multiple biological processes (Figure 1; STRING CONSORTIUM 2020, version 11.5). Previous studies have reported the presence of LGALS3BP as a component in the proteinaceous linker between somatic cell centrioles [29,30]. Furthermore, Fogeron et al. demonstrated deleterious effects on centrosome regulation when the protein was depleted using LGALS3BP-targeted small interfering RNA [29].

Depletion in HEK293 cells also led to multiple unequally sized centrosome-like structures, indicating the vital role of this protein across cell types [29]. In certain rodent species, including the rat, the proximal centriole will disintegrate throughout spermatogenesis; however, retention of this centriole throughout sperm maturation has been confirmed using transmission electron microscopy across numerous mammalian species, including the domestic cat and tiger [31], bull [32], ram [33], rabbit and man [34]. Together, the proximal and distal centrioles in these species form the sperm centrosome located at the base of the sperm head [32,35]. After fertilization, the sperm centrosome serves as an organizing center for numerous microtubules that are collectively referred to as the sperm aster [4,36,37,38]. This aster guides the male and female pronuclei together (apposition) for the completion of pronuclear syngamy in a timely fashion, which will, in turn, ensure the success of early embryonic development [4,35,36,37,38,39]. Inversely, an immature centrosome results in a significantly smaller sperm aster and a delayed first mitotic division, which compromises the subsequent embryo formation [4]. We previously demonstrated in the domestic cat that epididymal EVs transfer key proteins to the sperm cells and contribute to the maturation and function of the centrosome, which results in improved embryonic development following in vitro fertilization (IVF) [25]. Notably, LGALSB3BP has not been identified as being present within the oocyte. Although it has been detected in other regions of the female reproductive tract such as the endometrium [40,41] or in the follicular fluid [42], it was not identified in the mouse oocyte proteome [43,44] nor in the proteomic analysis of domestic cat germinal vesicles [45]. This suggests that the protein is likely introduced in the oocyte only by the sperm cell upon fertilization. Interestingly, LGALS3BP has also been identified as a biomarker for male infertility. LGALS3BP was found to be overexpressed in azoospermic patients and under-expressed in patients exhibiting oligoasthenozoospermia [46]. Additionally, Freour et al. identified LGALS3BP as a key seminal plasma indicator of viable testicular sperm in patients with non-obstructive azoospermia [47]. 

Based on the above observations about LGALS3BP, as well as our former studies on the domestic cat model [25], we hypothesized that LGALS3BP might be one of the key proteins transferred to the maturing spermatozoa via epididymal EVs contributing to full sperm functionality (fertilizing ability and developmental potential). Using the domestic cat model, the objectives of the present study were to (1) detect the presence and characterize the transfer of LGALS3BP via EVs between the epididymis and maturing sperm cells and (2) demonstrate the impact of LGALS3BP transfer on sperm fertilizing ability and developmental potential. 

## 2. Results

### 2.1. Nanoparticle Tracking Analyses of the Extracellular Vesicles

**Figure 2:**
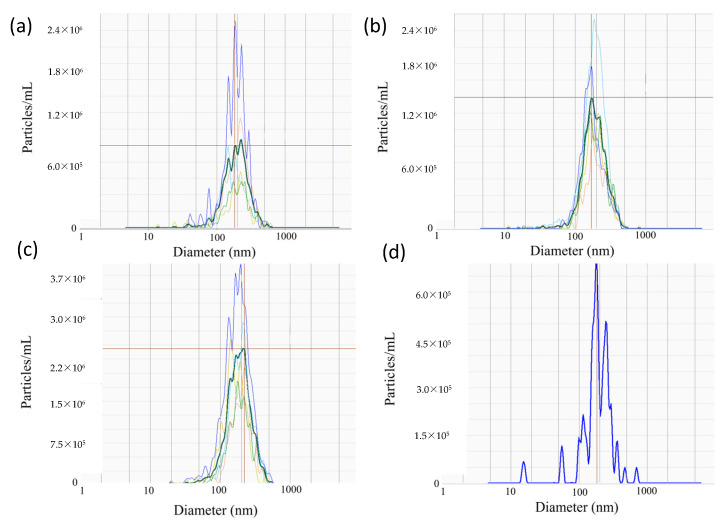
Representative spectra of EVs isolated from the (**a**) caput, (**b**) corpus, and (**c**) cauda of the epididymis (n = 5 males), as well as (**d**) whole epididymides (n = 1 pool of 5 males). Dark lines correspond to the segment average, whereas faint lines represent individual samples from which the average was derived. Red horizontal lines mark peak value of segment average.

Nanoparticle tracking analyses were performed to determine the size distribution of epididymal EVs (Figure 2). Overall, the average sizes of EVs were relatively consistent between the caput (264.14 ± 9.23 nm; mean ± SEM), corpus (271.18 ± 9.80 nm), and cauda segment (269.60 ± 10.33 nm), as well as the pooled sampled of whole epididymal EVs (285.8 nm; Figure 2).

### 2.2. Presence and Localization of LGALS3BP in Testicular Tissues, Extracellular Vesicles, and Sperm Cells 

**Figure 3:**
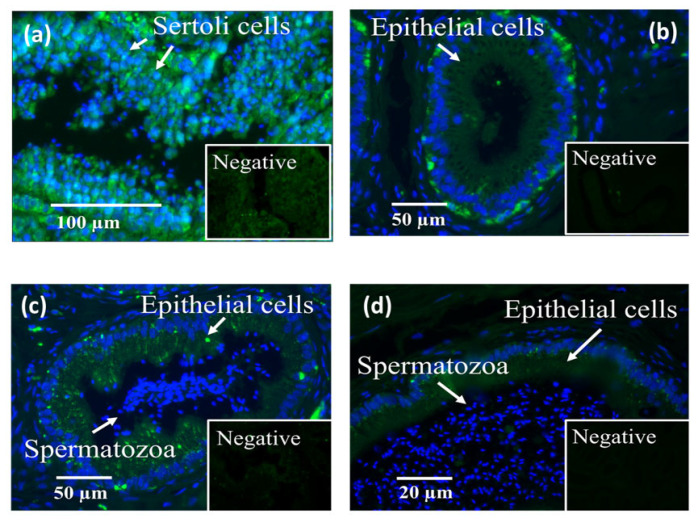
Localization of LGALS3BP using immunofluorescence (FITC; insets are negative controls) and nuclear chromatin counterstaining (Hoechst) in cross sections of (**a**) seminiferous tubules, (**b**) caput, (**c**) corpus, and (**d**) cauda of the epididymis (n = 5 males).

**Figure 4:**
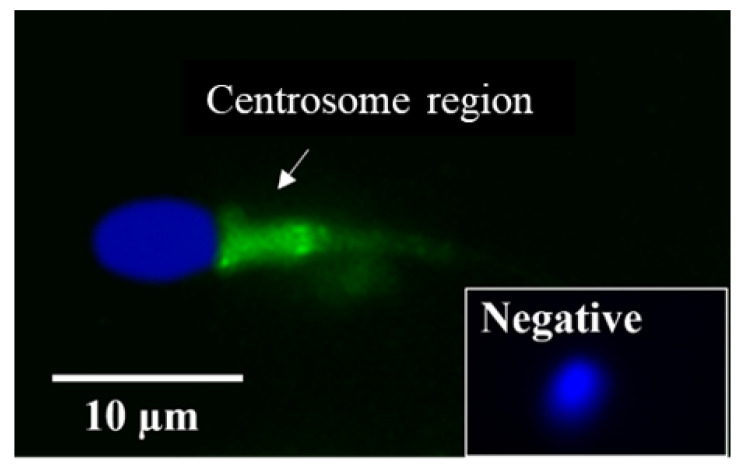
Localization of LGALS3BP using immunofluorescence (FITC; inset is negative control) and nuclear chromatin counterstaining (Hoechst) from a sperm cell isolated from the cauda of the epididymis.

**Figure 5:**
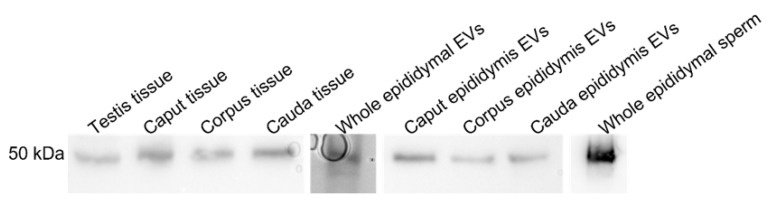
LGALS3BP detection using Western blot analysis in testicular tissues (n = 5 males), epididymal tissue (n = 5 males); pooled samples of EVs (n = 5 replicates; pools of 5 males each); epididymal EVs from caput, corpus, and cauda of the epididymis (n = 5 males); pooled samples of sperm cells from whole epididymis (n = 6 males).

**Figure 6:**
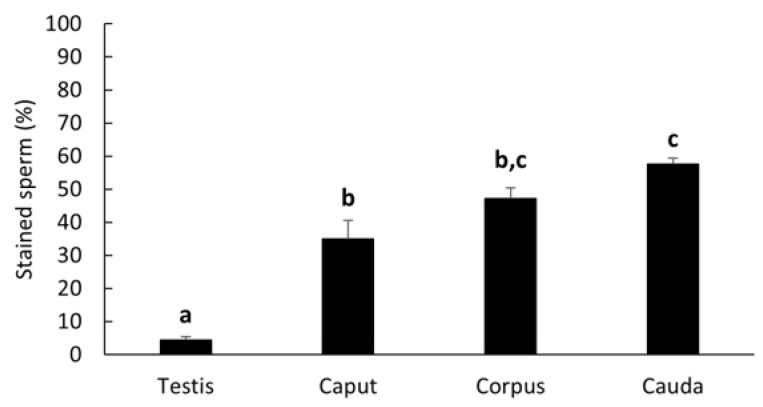
Percentages (mean ± SEM) of spermatozoa stained for LGALS3BP in the centrosome region from testicular tissue and each epididymal segment (n = 5 males). Bars with different letters significantly differ (*p* ≤ 0.001).

LGALS3BP was detected in the seminiferous tubules of the testicular tissue (Figure 3a), as well as in epithelial cells from the caput, corpus, and cauda of the epididymis (Figure 3b–d). LGALS3BP localization in the different segments of the epididymis appeared evenly distributed throughout the epithelial cells (Figure 3c,d). In sperm cells, LGALS3BP labeling was localized in the centrosome region (Figure 4). Western blot analysis, as well as mass spectrometry of whole epididymal tissue (Supplementary Files S1 and S2), confirmed the presence of LGALS3BP in tissues, EVs, and sperm cells isolated from the whole epididymis (Figure 5). Percentages of spermatozoa containing LGALS3BP increased progressively during the epididymal transit, with higher values (*p* < 0.0001) in sperm cells isolated from the cauda of the epididymis (57.6 ± 1.8%) compared to testicular sperm cells (4.4 ± 1.0%; Figure 6). 

### 2.3. Transfer of LGALS3BP from the Extracellular Vesicles to the Sperm Cells and Effects on Sperm Fertilizing Ability and Developmental Potential 

**Figure 7:**
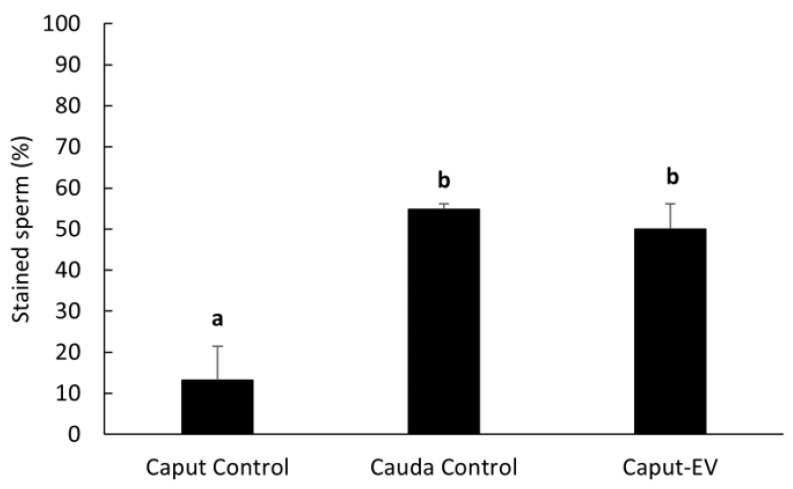
Percentages (mean ± SEM) of spermatozoa positively stained for LGALS3BP in the centrosome region. “Caput Control” spermatozoa were incubated for 3 h with plain buffer medium; “Cauda Control” spermatozoa were not exposed to EVs; “Caput-EV” spermatozoa were incubated for 3 h with EVs (n = 5 males per treatment). Bars with different letters significantly differ (*p* ≤ 0.01).

Percentages of sperm cells with LGALS3BP detected in the centrosome region significantly increased (*p* = 0.0016) in caput spermatozoa co-incubated with EVs (50.0 ± 6.2%) compared to the negative control incubated with plain buffer medium (13.3 ± 8.2%) and reached similar percentages as the cauda control (54.8 ± 1.4%; Figure 7). 

**Figure 8:**
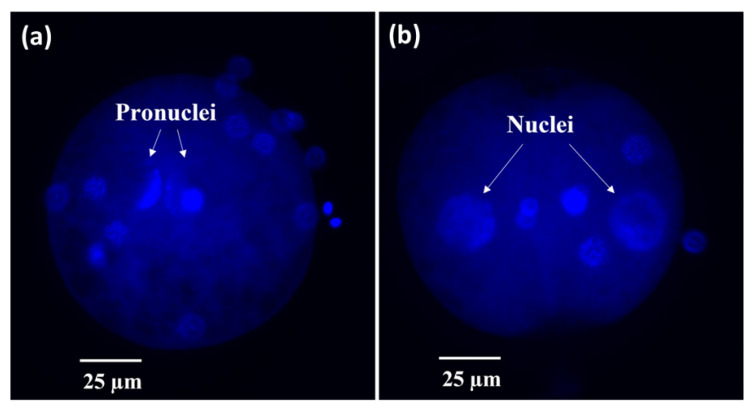
Representative micrographs of fertilized cat oocytes fixed at 17 h post-insemination and stained with Hoechst. (**a**) Fertilized oocyte or (**b**) 2-cell embryo.

**Figure 9:**
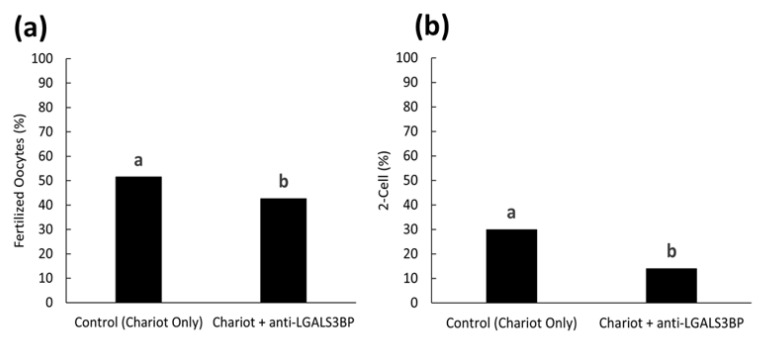
Proportions of (**a**) fertilized oocytes and (**b**) 2-cell embryos at 17 h post-insemination with oocytes previously treated with Chariot reagent alone (control Chariot only) or with Chariot- transfected EVs with anti-LGALS3BP (Chariot + anti-LGALS3BP). All oocytes were fertilized with untreated cauda spermatozoa. Bars with different letters significantly differ (*p* < 0.05).

**Figure 10:**
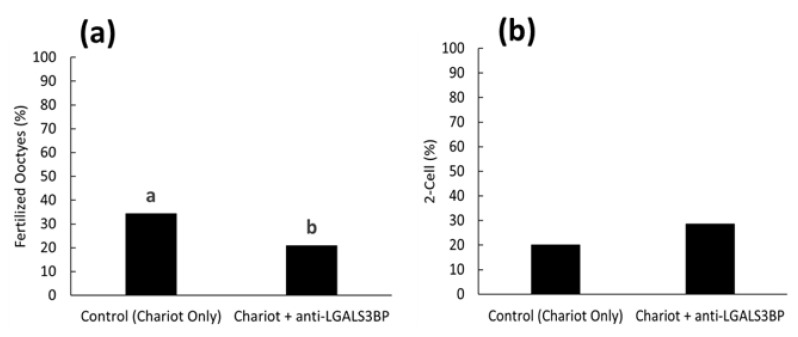
Proportions of (**a**) fertilized oocytes and (**b**) 2-cell embryos 17 h after insemination with caput spermatozoa previously treated with EVs transfected with Chariot reagent alone (control Chariot only) or with Chariot-transfected EVs with anti-LGALS3BP (Chariot + anti-LGALS3BP). Bars with different letters significantly differ (*p* < 0.05).

We then assessed the impact of inhibiting LGALS3BP (delivered by the sperm cell) on fertilization and the kinetics of the first cell cycle. First, the protein was inhibited by transfecting anti-LGALS3BP antibodies into oocytes prior to IVF with cauda spermatozoa. Percentages of metaphase II oocytes transfected with the Chariot reagent alone resulted in a higher percentage of fertilization compared to oocytes transfected with antibodies (51.5 and 42.7%, respectively; *p* < 0.05; Figure 8a and Figure 9a). Development to the two-cell stage using 17 h post-insemination was better in the control treatment compared to oocytes transfected with antibodies (29.9 and 14.0%, respectively; *p*< 0.05; Figure 8b and Figure 9b). We then explored if inhibiting the transfer of this protein to the sperm cell via EV would have similar deleterious effects. LGALS3BP was inhibited in the EVs via Chariot transfection before co-incubation with immature caput sperm cells, followed by IVF. Percentages of metaphase II oocytes fertilized by caput spermatozoa exposed to EVs transfected with Chariot alone was higher compared to sperm co-incubated with EVs transfected with antibodies (34.3 and 20.9%, respectively; *p* = 0.0395; Figure 8a and Figure 10b). There was no difference between treatment groups in the development to the two-cell stage using 17 h post-insemination (20.0 and 28.6%, respectively; *p* = 1.390; Figure 8b and Figure 10b).

## 3. Discussion

LGALS3BP was detected in the seminiferous tubules and epididymal epithelial cells. For the first time, this protein was observed in EVs secreted by the epididymal epithelium. We demonstrated that LGALS3BP was incorporated into the sperm cells via EVs and then localized in the sperm centrosome region. When LGALS3BP was inhibited in the oocyte during IVF with mature sperm cells, the results indicated the key role of LGALS3BP in promoting the sperm–oocyte interaction, as well as the capacity to complete a faster first cell cycle. When LGALS3BP was inhibited within epididymal EVs prior to incubation with immature sperm cells and IVF, lower percentages of fertilization further demonstrated the key role of EVs in the transfer of this vital protein to the spermatozoa. 

Nanoparticle tracking analyses characterized the EVs secreted by the epididymal epithelium. The average size of the epididymal EVs was approximately 285.8 nm, which is close to sizes in other species, including the Chinese hamster (range 50–250 nm, [48]), mouse (50–150 nm, [8]), and bull (25–300 nm, [49]). While LGALS3BP has been previously reported to weigh approximately 65–90 kDa in the other species, our analyses in the cat revealed a smaller protein weighing approximately 55 kDa in all sample types. This difference in molecular weight may be due to the post-translational modification of the protein specific to the domestic cat or possible proteolytic cleavage during sample processing. 

LGLAS3BP was detected and localized in the seminiferous tubules and epithelial cells of the epididymis, with the same patterns as in somatic cells from other species [50]. It was also observed at the sperm centrosome region, as in the rat model [29]. However, for the first time, we showed that the proportion of sperm cells with LGALS3BP localized at the centrosomal region increased throughout epididymal transit. The proportion also increased following exposure of immature spermatozoa with EVs, indicating the role of these vesicles in transferring the protein. It should be noted that there was a slight difference between the percentages of caput sperm cells containing LGALS3BP in Figure 6 and Figure 7. This is likely due to natural variations between the males used for each study. 

Inhibiting LGALS3BP via Chariot transfection of oocytes with anti-LGALS3BP antibodies during IVF resulted in decreased fertilization. Previous reports have demonstrated that depleting LGALS3BP in somatic cells results in diminished centrosomal functions with subsequent anastral spindle organization, as well as the absence or reduction in the size of the centrosome [29]. While the function of LGALS3BP in the sperm cell has not yet been directly analyzed in any species to date, our former studies in the domestic cat had observed similar results when assessing the effects of malformed or immature sperm centrosomes on the production of the sperm aster following fertilization [4]. Similarly, studies assessing sperm centrosome-related male infertility have also reported anastral spindle organization and decreased embryo survival [51,52]. Another study found that this infertility could be overcome by injecting oocytes with spermatozoa that displayed properly aligned head–tail junctions [53]. Decreased embryonic development to the two-cell stage using 17 h post-insemination in the anti-LGALS3BP-transfected oocytes during IVF reemphasized a disruption to the normal centrosome function and subsequent delay in the first mitotic cleavage. As previously reported, a delayed development to the two-cell stage at the 17 hr time point is related to impaired sperm centrosomal function [4,25]. 

We previously demonstrated that EVs transfer numerous key proteins to the developing spermatozoa, which aids in the centrosomal maturation and ability to successfully coordinate the first mitotic cell division [25,26]. Exposing immature caput sperm cells to EVs also previously resulted in improved embryonic development to the morula and blastocyst stage following IVF [25]. In the present study, we observed deleterious effects on fertilization when LGALS3BP was inhibited within EVs prior to incubation with immature caput sperm cells, followed by IVF. These results are consistent with the negative effects observed when LGALS3BP was first inhibited within the oocyte prior to fertilization by untreated cauda spermatozoa. These data further demonstrate the vital role of EVs in transferring essential proteins from the epididymal epithelium to the maturing spermatozoa. However, development to the two-cell stage using 17 h post-insemination was not decreased in the EV Chariot–control treatment. This may be due to compensatory mechanisms within the oocyte cytoplasm following fertilization or the insufficient inhibition of the LGALS3BP protein by the Chariot reagent throughout the duration of the experiment. As the EVs and caput spermatozoa were subject to a greater degree of handling and manipulation via centrifugations and incubations, it is possible that some protein molecules may have escaped sufficient inhibition at the point in which IVF was performed. 

As we recently reviewed, there are still many unknows regarding epididymal EVs in many different species [54]. However, the present findings improve our understanding of a key sperm protein carried by EVs that is involved in successful sperm–oocyte interactions and fast completion of the first cell cycle. Since previous studies reported the use of LGALS3BP as a biomarker for other types of male infertility [46], incorporating the use of EVs as a supplement may improve the success of treatments for this type of condition. In sum, deciphering the key roles of LGALS3BP could lead to new approaches to enhance or even control fertility in clinical settings for animals and humans. 

## 4. Materials and Methods

All chemicals and reagents were purchased from Sigma Chemical Company (St. Louis, MO, USA), unless otherwise stated. 

### 4.1. Tissue and Extracellular Vesicle Collections

Domestic cat testes and ovaries from adult individuals (>1 year old) were supplied to our laboratory by local veterinary clinics. All cats were healthy and did not have any history of chronic pathology or reproductive issues. This study did not require the approval of the Animal Care and Use Committee of the Smithsonian National Zoo and Conservation Biology Institute because cat gonads were collected as byproducts from owner-requested routine orchiectomies or ovariohysterectomies. Male and female tracts were transported and stored in phosphate buffered saline (PBS) at 4 °C for less than 6 h until processing. Spermatozoa were collected separately from caput and cauda of the epididymis by slicing tissue using scalpel blade in Hepes–Hams medium F-10 (Irvine Scientific, Santa Ana, CA, USA) (25 mM Hepes, 1.0 mM pyruvate, 2.0 mM L-glutamine, 100 IU/mL penicillin, 100 μg/mL streptomycin, and 5% fetal calf serum), followed by 5–10 min incubation at room temperature to allow sperm cells to seep out.

Luminal fluids containing EVs were recovered in a similar manor except placed into plain Hepes–Ham medium F-10 (Irvine Scientific), without fetal bovine serum from pools of n = 5 males, each as previously described [25,26]. Briefly, cell debris was discarded from the supernatant using a series of centrifugations at 700× *g* for 10 min and 3000× *g* for 10 min at room temperature. The EV fraction was then isolated from the remaining supernatant using ultracentrifugation at 100,000× *g* for 2 h at 4 °C and resuspended in fresh Ham’s F-10 media (Irvine Scientific). Aliquots of EV samples were stored at −20 °C until further processing. Successful isolation of EVs was previously confirmed via observations performed using a transmission electron microscope (Zeiss 10 CA Transmission Electron Microscope, Dublin, CA, USA) at the University of Maryland Laboratory for Biological Ultrastructure, College Park, MD, USA [26]. All relevant data from collection may also be found in the EV-TRACK knowledgebase (EV-TRACK ID: EV200074, https://evtrack.org/, accessed on 25 November 2022). It should be noted that this study used the operative term, “Extracellular Vesicle” (EV), in accordance with the guidelines set forth by the International Society for Extracellular Vesicles [55]. ISEV endorses the use of this term when defining an isolated sample that has not been further analyzed for EV subtypes (e.g., exosome, microvesicle, apoptotic vesicle, etc.). 

### 4.2. Characterization of Extracellular Vesicles using Nanoparticle Tracking Analysis

EV samples were isolated from each consecutive segment of the epididymis (caput, corpus, and cauda) from individual males (n = 5 males total) and whole epididymides of 5 males pooled together (n = 1 pooled sample). Nanoparticle tracking analysis (NTA) was performed using Cell Guidance Systems (St. Louis, MO, USA). Upon receipt of the samples, they were stored at −80 °C until the day of analysis. Samples were collected in dry ice, and each sample thawed individually at room temperature for analysis. Once thawed, samples were vortexed and sonicated in a water bath for 3 min. Samples were then diluted in 0.2 µm sterile filtered deionized water to achieve the desired dilution and placed into a well on a 24-well plate and aspirated in a 1 mL syringe before being dispensed into the Particle Metrix Zetaview for size analysis.

### 4.3. Tissue Processing and Immunostaining

Testicular and epididymal tissue samples were fixed in 4% parafomaldehyde, dehydrated, embedded in paraffin, and sectioned at a thickness of 5 μm. Samples then underwent 10 min antigen retrieval at 95 °C (10 mM citric acid, 3 mM methylenediaminetetraacetic acid supplemented with 1% Triton-X), and permeabilized with 0.1% Triton X-100 in PBS (PBS-T) for 3 min. The non-specific antigenic sites were blocked in 5% bovine serum albumin in PBS (1 h, room temperature) and incubated with anti-LGALS3BP (1:100) antibodies overnight at 4 °C in a humidified chamber. After washing (5 min each) in PBS twice and PBS-T once, samples were incubated with secondary antibodies labeled with a fluorescent probe for 1 h at 37 °C (goat anti-mouse 1:100) before observation under a microscope fitted with epifluorescence (Olympus BX41, Bartlett, TN, USA). Negative control treatments were also included in which primary antibody was omitted, and samples were incubated with the secondary fluorescent antibody.

### 4.4. Western Blot Analysis and Mass Spectrometry Confirmation

Testicular and epididymal tissue samples were isolated and homogenized in Tween-20 lysis buffer (150 Mm sodium chloride, 50 mM Trizma base, 1% Tween-20) and centrifuged at 14,000× *g* for 15 min at room temperature for further analyses via Western blot. EV samples were isolated in plain Ham’s F-10 buffer (Irvine Scientific), while sperm samples were isolated in PBS. All samples were diluted with SDS loading buffer (Boston BioProducts, Milford, MA, USA) and incubated at 95 °C for 10 min. Samples were then separated by one-dimensional electrophoresis (Bio-Rad 4–15% Mini-PROTEAN TGX Gel, Hercules, CA, USA) with tissue, EV, and sperm samples separated based on equivalent quantity of proteins (30 μg total protein per lane as determined by Bio-Rad assay kit using bovine serum albumin as standard). Proteins were transferred to a nitrocellulose membrane (Bio-Rad) and blocked for 1 h at room temperature in 1× Tris-buffered saline (154 mM Trizma HCl, 1 M sodium chloride supplemented with 1% Tween-20, and 7% skim milk powder). Membranes were then incubated overnight at 4 °C with anti-LGALS2BP (1:1000) and for 1 h at room temperature with goat anti-mouse secondary antibody coupled with horseradish peroxidase (1:1000) in 1× Tris-buffered saline supplemented with 1% Tween-20 and 5% skim milk powder, followed with Clarity Western ECL substrate (Bio-Rad) and imaged with ChemiDoc XRS imaging system (Bio-Rad) (Figures S1–S4 in Supplementary File S1). Results were also confirmed using gel digest analysis via liquid chromatography–mass spectrometry (LC-MS/MS) with a Waters nanoACQUITY HPLC system interfaced to a Thermo Fisher Q Exactive at MS Bioworks, USA (row 150 in Table S1; Supplementary File S2).

### 4.5. Oocyte Collection, Antibody Transfection, and In Vitro Fertilization

#### 4.5.1. Collection of Oocytes and In Vitro Maturation

Grade I immature oocytes were identified as having homogeneous dark cytoplasm and several layers of compacted cumulus cells and were isolated after slicing ovaries in dissecting medium (Eagle MEM with Hank’s balanced salt—Gibco Laboratories, Bethesda, MD, USA supplemented with 100 IU/mL penicillin, 100 µg/mL streptomycin, 10 mM HEPES, and 0.1% bovine serum albumin); lower quality oocytes were not used. Selected cumulus-oocyte complexes (COCs) were then cultured for 26 h at 38.5 °C in 5% CO_2_ in in vitro maturation (IVM) medium composed of SAGE blastocyst medium (Pasadena, CA, USA), supplemented with 0.01% (*v/v*) FSH and 0.01% LH (National Hormone and Pituitary Program, Rockville, MD, USA) as previously reported [56]. 

#### 4.5.2. Oocyte Antibody Transfection and In Vitro Fertilization Using Cauda Spermatozoa

After 26 h incubation, LGALS3BP protein inhibition was achieved by transfecting anti-LGALS3BP antibodies into COCs as previously described [57,58], using the Chariot peptide nanoparticle transfection reagent (Active motif, Carlsbad, CA, USA). Antibody-Chariot complexes were made according to manufacturer’s instructions. Briefly, 2 µL of Chariot was added to 50 µL of H_2_O with 1.5 µg of antibody in 50 µL of PBS for 30 min at room temperature. The assembled complexes were added to the COCs in IVM medium and were incubated at 38.5 C in 5% CO_2_ for 4 h then rinsed three times in SAGE blastocyst media prior to IVF. Sperm cells were isolated from the cauda of the epididymis and incubated in base medium, without EVs. Spermatozoa were then centrifuged at 300× *g* for 8 min and resuspended in Sage blastocyst medium (concentration = 5 × 10^6^ motile sperm/mL) prior to IVF. Oocytes were inseminated with sperm cells in 50-µL microdrops of SAGE blastocyst medium and incubated for 17 h at 38.5 °C in 5% CO_2_ before fixation in 4% paraformaldehyde overnight at 4 °C. 

### 4.6. Extracellular Vesicle Transfection and In Vitro Fertilization Using Caput Spermatozoa

Functional effects of EV exposure on immature sperm cells were analyzed using EV samples isolated from the entire tract. EVs were collected from whole epididymides (pools of 5 males). Protein inhibition was achieved by transfecting anti-LGALS3BP antibodies into EVs using the Chariot peptide nanoparticle transfection reagent (Active motif). Antibody-Chariot complexes were made according to manufacturer’s instructions. Briefly, 2 µL of Chariot was added to 50 µL of H_2_O with 1.5 µg of antibody in 50 µL of PBS for 30 min at room temperature. The assembled complexes were incubated with EVs in plain base medium (Ham’s F-10, Irvine Scientific; EVs = 4 µg/mL total protein) at 38.5 C in 5% CO_2_ for 4 h, followed by centrifugation at 16,000× *g* for 10 min and resuspended in fresh Hepes–Hams medium F-10 (Irvine Scientific). Spermatozoa were isolated from the caput of the epididymis and centrifuged at 300× *g* for 8 min and reconstituted with Hepes–Hams medium F-10 (Irvine Scientific) prior to EV incubation (concentration = 50 × 10^6^ sperm/mL). As previously reported [25,26], spermatozoa were then co-incubated with treated EV for 1 h 15 min, centrifuged once more at 300× *g* for 8 min, and resuspended in SAGE blastocyst medium (Cooper Surgical, Trumbull, CT, USA) prior to IVF. Oocytes were inseminated with sperm cells into 50-µL microdrops of SAGE blastocyst medium and co-incubated for 17 h at 38.5 °C in 5% CO_2_ before fixation in 4% paraformaldehyde overnight at 4 °C.

### 4.7. Staining and Observations of Oocytes and Embryos

The same staining protocol was used following both IVF experiments mentioned above. After three washings in medium (PBS supplemented with 0.5% Triton-X and 2% fetal bovine serum), non-specific binding sites were blocked in saturation medium (PBS supplemented with 20% fetal calf serum and 0.5% Triton-X) for 1 h at 38.5 °C. Samples were then incubated with Hoechst staining solution (1 µg/mL) for 10 min, at room temperature, before observation under a microscope fitted with epifluorescence (Olympus BX 41). Percentages of fertilized oocytes (presence of 2 pronuclei) and development to the 2-cell stage was recorded at 17 h post-insemination. Spot Basic 5.1 software (Diagnostics Instruments) was used to capture images of oocytes and embryos. As previously reported, higher proportions of 2-cell stage at the 17 hr time point indicated a faster first cell cycle because of functional sperm centrosomes [4,25].

### 4.8. Experimental Design and Statistical Analysis

In Study 1 (Figure 2), EV nanotracking analyses were performed to better characterize the vesicles isolated from the epididymal tract. EV samples were isolated from each consecutive segment of the epididymis from individual males (caput, corpus, and cauda; n = 5 males total; n = 5 replicates total, one male per replicate) and from whole epididymides of n = 5 males pooled together (n = 1 pooled sample, n = 1 replicate). In Study 2a (Figure 3 and Figure 4), immunostaining of testicular and epididymal tissues was performed to characterize the expression of LGALS3BP and its localization (samples were isolated from n = 5 males for a total of n = 5 replicates, one male per replicate). Then, Study 2b (Figure 5) focused on the LGALS3BP detection in the epididymal EVs via Western blot analyses. For these assays, EVs were collected separately from each consecutive segment (caput, corpus, and cauda) from n = 5 males (n = 5 replicates total, one male per replicate) as well as from the whole epididymis of n = 4 replicates (pools of 5 males per replicate). In Study 2c (Figure 6), presence of LGALS3BP in the developing spermatozoa was examined (sperm samples were isolated from each separate segment of n = 5 males for a total of n = 5 replicates, one male per replicate). In Study 3 (Figure 7), sperm samples were isolated from the caput and cauda from the epididymis of n = 6 males (n = 6 replicates total, one male per replicate) to assess the uptake of LGALS3BP following exposure to EVs. Study 4 focused on IVF analyses using the Chariot transfection reagent to assess the impact of inhibiting LGALS3BP on fertilization and early embryo development. In Study 4a (Figure 8 and Figure 9), the effects of inhibiting LGALS3BP were analyzed by transfecting oocytes prior to IVF. Treatment groups included oocytes transfected with anti-LGALS3BP antibodies, as well as transfection with just Chariot transfection reagent as control prior to IVF. Untreated cauda spermatozoa were collected from n = 12 males for embryonic development analyses following antibody transfection (n = 387 oocytes total; n = 12 replicates total for study, one male per replicate). In Study 4b (Figure 10), LGALS3BP was inhibited in the EVs via Chariot transfection prior to incubation with immature caput sperm cells, followed by IVF. We then examined the influence on subsequent fertilization and early embryo development. Caput spermatozoa were collected from n = 6 males for embryonic development analyses following antibody transfection (n = 140 oocytes total; n = 6 replicates total for study, one male per replicate).

Statistical analyses were conducted via GraphPad Prism software version 6. Differences between treatment groups for all analyses, except IVF analyses, were completed via analysis of variance (ANOVA) with results further compared via protected Tukey’s test, blocking for individual variation. In Study 4 (Figure 9 and Figure 10), numbers of oocytes that were available for each replicate were very variable. Data from all replicates were therefore pooled to calculate a single proportion. Data were then compared using chi-square goodness-of-fit.

## Data Availability

All relevant data from EV collection may also be found in the EV-TRACK knowledgebase (EV-TRACK ID: EV200074, https://evtrack.org/, accessed on 25 November 2022).

## Supplementary Materials

The supporting information can be downloaded at: https://www.mdpi.com/article/10.3390/ijms24043077/s1.
