# Supplementary material for: Transfer of Galectin-3-Binding Protein via Epididymal Extracellular Vesicles Promotes Sperm Fertilizing Ability and Developmental Potential in the Domestic Cat Model
Source: Int J Mol Sci. 2023 Feb 4;24(4):3077. doi: 10.3390/ijms24043077 (PMC9966717; doi:10.3390/ijms24043077)
Supplement: Supplementary file 1 [file ijms-24-03077-s001.zip › Rowlison Supplemental File S1.pdf]

Supplementary File S1

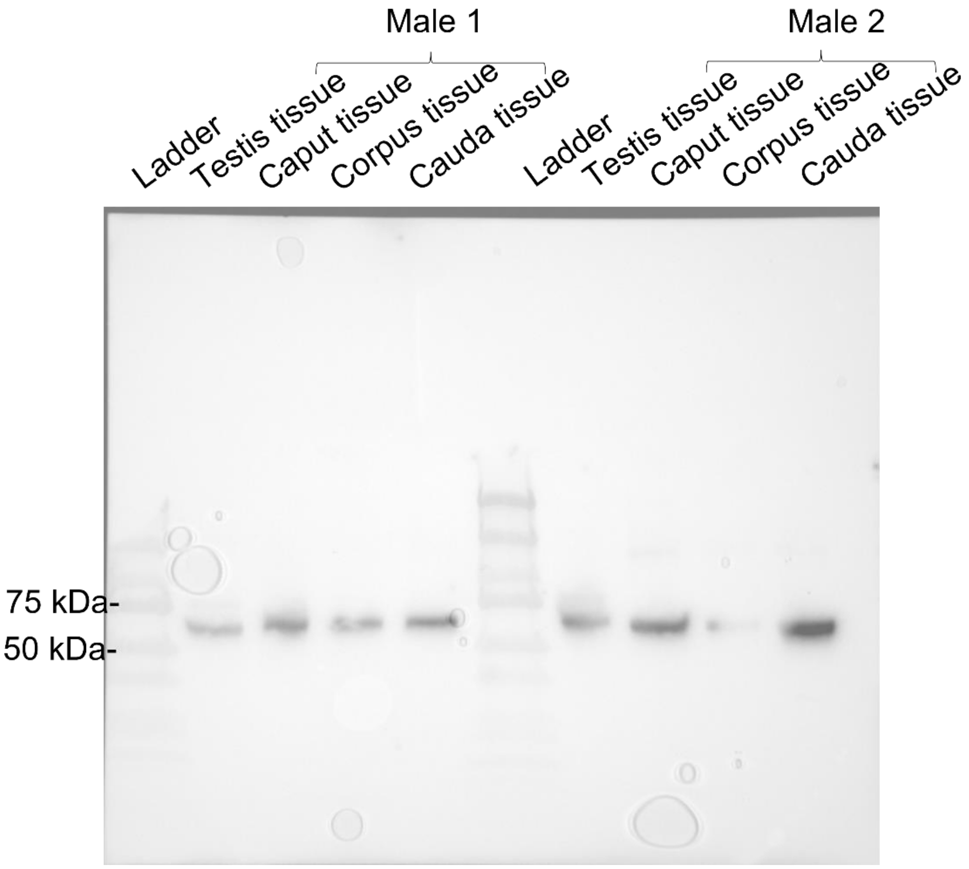

Figure S1. Example of LGALS3BP detection by Western Blots analysis in testicular and epididymal tissues.

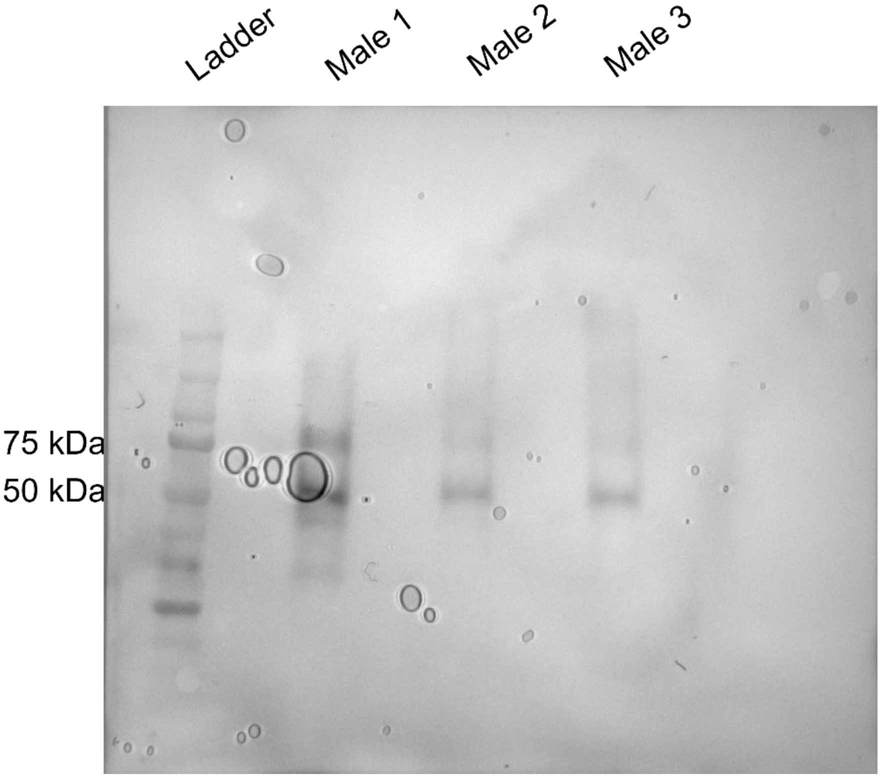

Figure S2. Example of LGALS3BP detection by Western Blots analysis in EVs isolated from whole epididymides.

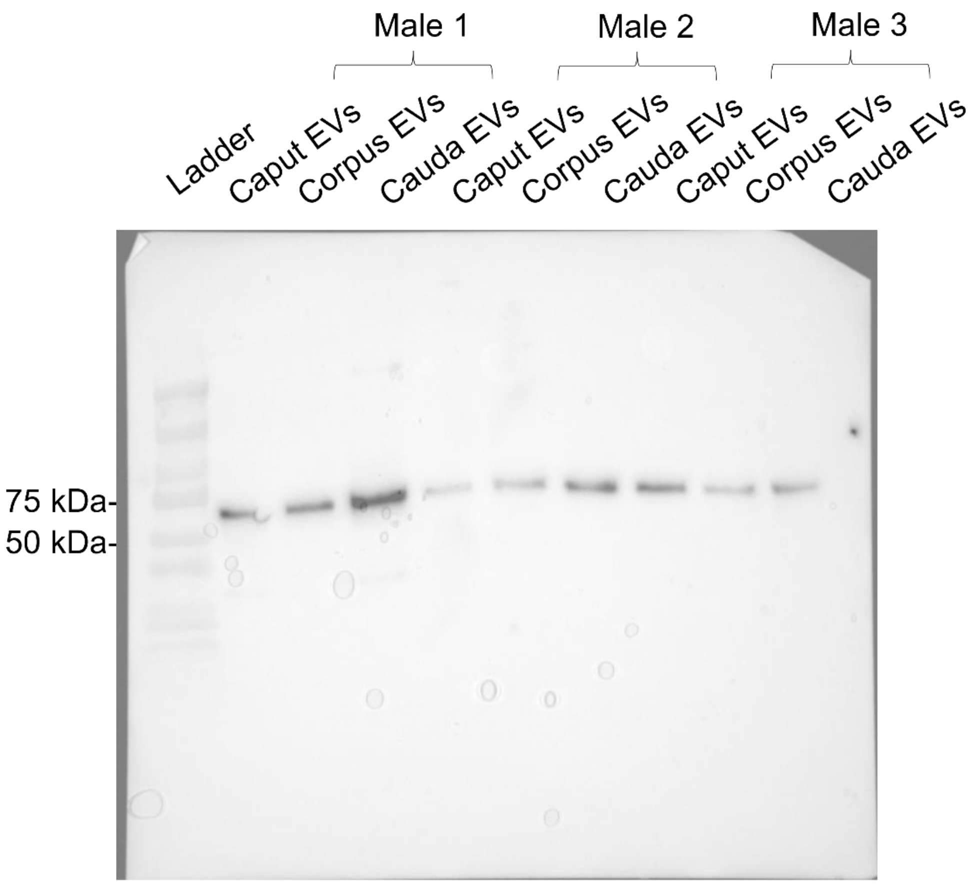

Figure S3. Example of LGALS3BP detection by Western Blots analysis in EVs isolated from each epididymal segment.

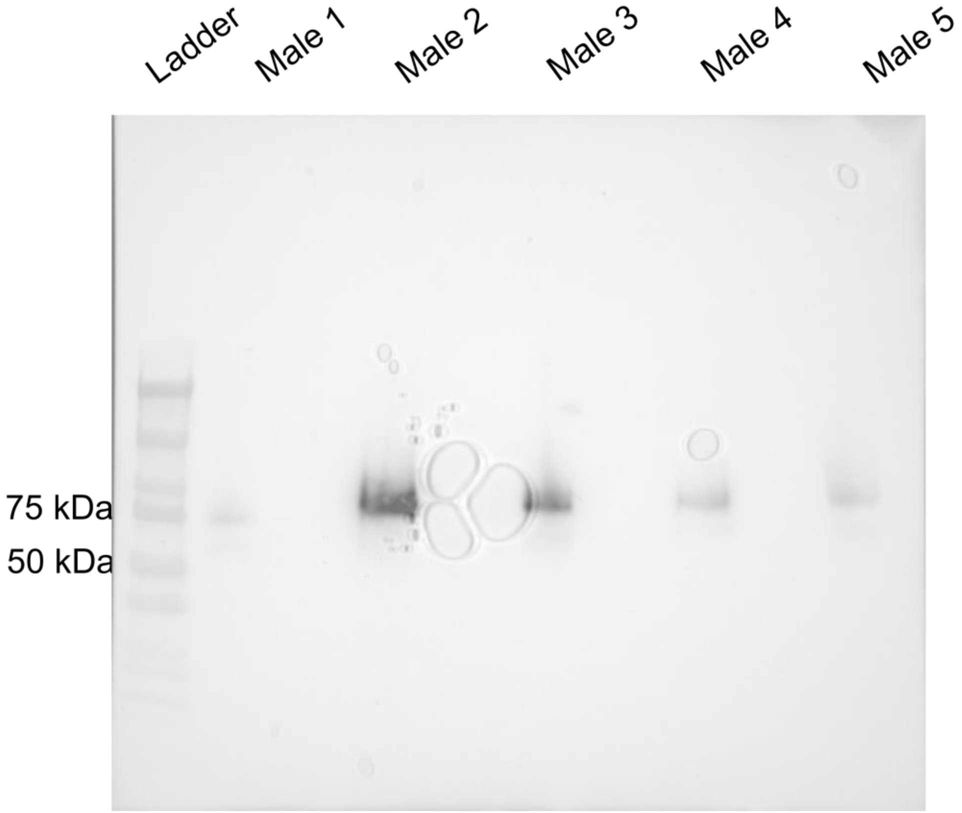

Figure S4. Example of LGALS3BP detection by Western Blots analysis in sperm cells isolated from whole epididymides.
